# Expanding Bicycle Infrastructure to Promote Physical Activity in Hidalgo County, Texas

**DOI:** 10.5888/pcd16.190125

**Published:** 2019-09-12

**Authors:** Evelia C. Castillo, Monica Campos-Bowers, Marcia G. Ory

**Affiliations:** 1 Texas A & M University, Health Science Center, South Texas Center-McAllen Campus, McAllen, Texas; 2 Texas A & M University, Health Science Center, Department of Environmental and Occupational Health, College Station, Texas; 3 Texas A & M University, Center for Population Health and Aging, College Station, Texas

## Abstract

The role of the built environment as both an asset and a barrier in promoting physical activity is well documented. However, literature on the role of communities in catalyzing policy, systems, and environmental (PSE) change to address gaps in the built environment is scant. We describe a community-driven PSE intervention, resulting in expanded bicycle infrastructure and physical activity opportunities in a South Texas border community. Funded through the Centers for Disease Control and Prevention, the Working on Wellness project engaged community-based coalitions in efforts to increase opportunities for physical activity in Hidalgo County, Texas. Coalitions collaborated with the city of Weslaco to install bicycle lanes and with the Hidalgo County Metropolitan Planning Organization to establish a countywide Bicycle Friendly Business program. Community-driven PSE interventions can be effective public health strategies in creating long-term sustainable solutions that address environmental determinants of obesity.

**Summary**

**What is already known on this topic?**

Although policy, systems, and environmental (PSE) strategies can expand access to healthy living infrastructure, public health programs have not traditionally incorporated PSE components in community health interventions.

**What is added by this report?**

Community coalitions collaborated to design and implement PSE-focused interventions resulting in expanded bicycle infrastructure and sustainable public health activities in Hidalgo County, Texas.

**What are the implications for public health practice?**

Involving community members and building their capacity to implement PSE interventions is necessary to address systemic barriers to public health. Health programming that is community driven and incorporates PSE strategies can maximize impact, reach, and sustainability of public health interventions.

## Background

### Demographics

Located along the southern United States border with Mexico, Hidalgo County is part of a 4-county region known as the Rio Grande Valley. The county’s 860,861 residents are predominantly Hispanic (92%), and nearly one-third (29.5%) live in poverty (1). Educational attainment lags behind both the state and nation. Only 63.7% of adults aged 25 years or older have a high school diploma or higher, compared with 82.8% at the state level and 87.3% at the national level (1). This difference is meaningful because low socioeconomic status is associated with poor health outcomes (2).

### Obesity and health outcomes

Obesity is a persistent public health challenge in Hidalgo County. County statistics in 2019 show that 1 of every 3 people is obese (3). High rates of obesity contribute to health disparities in the region. Approximately 27% of adults have diabetes and another 32% have prediabetes (4). Diabetes can lead to serious health complications over time, including cardiovascular disease, vision loss, kidney failure, and limb amputation (5).

Regular physical activity can help prevent obesity and associated chronic diseases. However, lack of safe spaces in the built environment — physical structures built or designed by humans (ie, roads, buildings, sidewalks, and bike paths) (6) — can limit opportunities for people to be physically active. Over one-fifth (21%) of Hidalgo County residents report no physical activity (3). Sedentary lifestyles can increase the risk of developing chronic diseases such as obesity, diabetes, and heart disease (3).

### Working on Wellness (WoW) program

Established in 2015, the WoW program aims to improve health outcomes by addressing environmental and policy determinants of obesity. The WoW program team used the Hexagon Tool (7), a readiness assessment instrument that examines 6 factors (need, fit, resources, evidence, readiness, and capacity) to determine intervention communities in Hidalgo County. On the basis of this initial assessment, Peñitas, San Carlos, South McAllen, and Weslaco were identified as the intervention communities. Program staff at Texas A & M University’s AgriLife Extension Service and the School of Public Health partnered with community members to establish coalitions in each of the intervention communities. Policy, systems, and environmental (PSE) intervention strategies aim to enhance or create structures in which people live, work, and play with the goal of creating long-term, sustainable changes that support healthy living. WoW coalitions collaborated with private and public sector partners to design and implement PSE change interventions focused on improving healthy behaviors by enhancing physical activity and healthy food access.

### Partnerships and collaborations

Complementing a community-driven approach to local decision making about PSE strategies, AgriLife Extension Service and the School of Public Health provided support at the outset to help establish community coalitions and funding to implement interventions. Additionally, WoW staff members provided technical assistance throughout the grant period.

In our example intervention community of Weslaco, Weslaco High School, the South Texas Juvenile Diabetes Association, and bicycling advocates were key members of the Weslaco coalition. These coalition members were instrumental in obtaining buy-in from city leaders, including the mayor, the city manager, and the parks and recreation director. Coalition members also helped secure additional grant and in-kind resources from city government and private sector partners.

The Hidalgo County Metropolitan Planning Organization (HCMPO) collaboration was key in advancing interventions with an active transportation focus. HCMPO staff created the Bike Friendly Business program, and in partnership with WoW, implemented the program countywide. The HCMPO’s Bicycle Pedestrian and Advisory Committee also served as a platform to collaborate with transportation experts on active living planning for the region.

## Expanding Bicycle Infrastructure

A community participatory approach was key in building trust and meaningful partnerships that facilitated community buy-in and leveraged partner resources. In collaboration with community members, WoW staff members conducted a baseline needs assessment in 2015 to assess the built environment. Findings indicated limited active living infrastructure to support physical activity for community members. This process also identified people to help establish community coalitions in each intervention strategy. Additionally, the needs assessment helped tailor technical assistance programming to build the capacity of community coalitions and partners to develop, implement, and sustain PSE interventions.

On the basis of an asset mapping activity and findings from the needs assessment, each community coalition worked toward identifying and selecting high-priority projects for interventions. WoW staff members facilitated this process by applying the Strategic Doing (8) approach, developed by the Purdue Agile Strategy Laboratory. Strategic Doing facilitates action-oriented collaborations by systematically approaching an opportunity and creating a shared action plan for implementation that includes identifying key stakeholders, existing resources, and concrete next steps.

### Intervention selection and implementation

In the intervention community of Weslaco, coalition members prioritized street connectivity and expansion of bicycle infrastructure. The coalition identified Panther Loop, an informal path surrounding Weslaco High School, as a potential intervention site. The Family, Career and Community Leaders of America club at Weslaco High School completed an assessment of Panther Loop. During this time, coalition members also identified and mapped potential locations for bicycle lanes.

**Figure 1:**
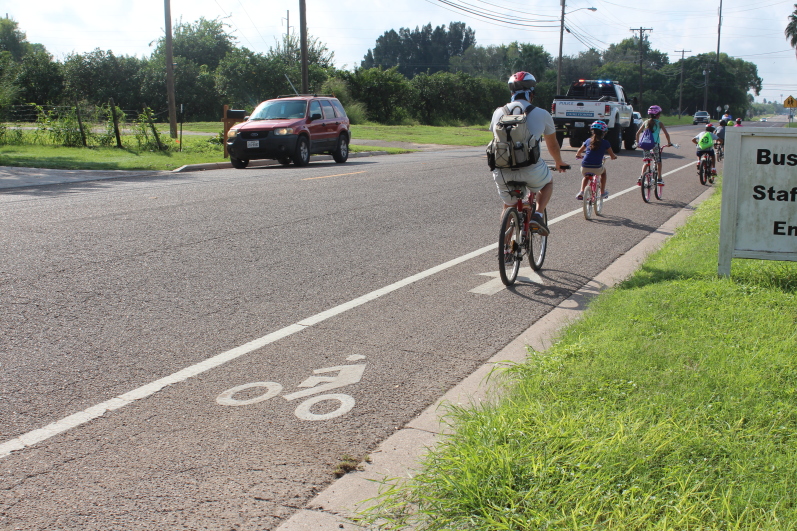
Weslaco Bikearoos bike ride, Weslaco, Texas, October 2017. Photo produced by Evelia Castillo.

The coalition presented the Panther Loop and bicycle lane recommendations to the mayor, the city manager, and the parks and recreation director. After discussing available resources, potential reach, and the feasibility of project implementation and sustainability, the coalition and the city agreed to move forward with the bicycle lane project. Installing bicycling lanes increases ridership even in communities with no cycling culture (9). This holds true even when controlling for other variables that might influence transportation modes including land use, climate, socioeconomic factors, gasoline prices, public transport supply, and cycling safety (9). With financial support from the WoW coalition, the city installed 5 miles of bicycle lanes in September 2016. Local partners also created the Weslaco Bikearoos program to educate community members on bike safety and hosted group rides along the bicycle lanes. Through the Weslaco Bikearoos, quarterly bike rodeos and group bike rides began in February 2017 (Figure 1). During bike rodeos, children learn about bike safety and practice their new skills on an obstacle course and on a 3-mile group ride afterwards. Teens and adults participate in bike rides twice a week along the bicycle lanes.

**Figure 2:**
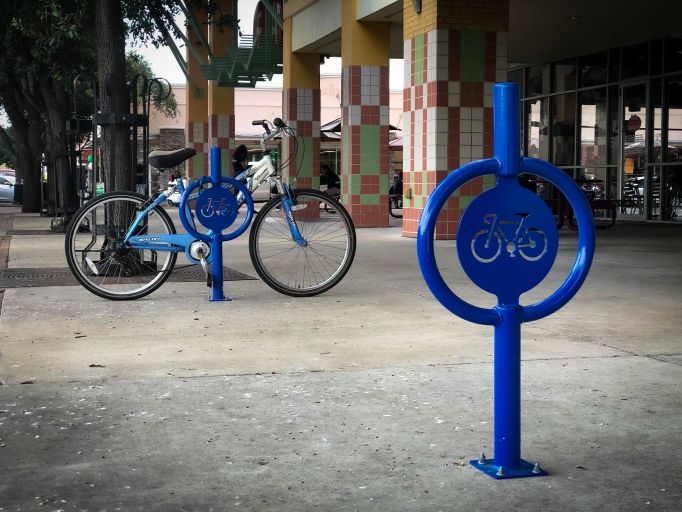
Bicycle Friendly Business, representing activities outside of the 4 intervention communities, McAllen, Texas, June 2019. Photo by Robert DeLeon. [A text description of this figure is also available.]

In response to findings that indicated a lack of infrastructure for active living in the county, WoW staff and coalition members joined the HCMPO’s Bicycle Pedestrian and Advisory Committee to engage transportation experts in WoW interventions. This engagement resulted in collaboration with the HCMPO to implement the Bicycle Friendly Business program. WoW provided bicycle racks to incentivize businesses to join the program, and coalition members helped with recruitment (Figure 2). Fifty-one businesses in the county have joined the Bicycle Friendly Business program, supporting active living by providing bicycle parking, free water refill stations, restrooms, bicycle repair kits, and special discounts for cyclists. Bicycle Friendly Business members installed 57 bicycle racks to accommodate 190 bicycles across multiple locations in 6 cities throughout Hidalgo County.

Coalition members collaborated with city leaders in Weslaco and the HCMPO to formulate policy-focused interventions. In Weslaco, the coalition actively participated in the development of a master trails plan for both bicycling and hiking (10). Coalition members attended 3 workshops hosted by Weslaco Parks and Recreation to provide input and recommendations in spring 2017. Weslaco commissioners officially adopted the Weslaco Master Trails Plan in June 2017. This systems-focused plan creates an “integrated seamless transportation and recreation framework to facilitate hiking and biking as a viable transportation alternative throughout Weslaco” (10). Coalition members continue to advocate for implementation of the Weslaco Master Trails Plan by engaging with city leaders and attending city commission meetings. In 2018, the city installed an additional one-half mile of bicycle lanes.

In collaboration with HCMPO staff, the Bicycle Pedestrian and Advisory Committee submitted an application to attend the 4th Annual Walkability Action Institute, hosted by the National Association of Chronic Disease Directors in Decatur, Georgia. An interdisciplinary group of Bicycle Pedestrian and Advisory Committee members — including a coalition member from Weslaco and coalition partners from McAllen and San Carlos — was selected to attend the workshop. The team developed an action plan with PSE outcomes to improve walkability in Hidalgo County. The Bicycle Pedestrian and Advisory Committee formally adopted the Hidalgo County Walkability Team Action Plan in August 2018. WoW staff and coalition partners collaborated with the HCMPO to implement the plan. This plan included organizing a Complete Streets Workshop, delivered in October 2018.

## Implications for Public Health

Building healthy communities calls for transformative changes that address systemic barriers to public health. WoW efforts demonstrate that such change is possible through PSE interventions that apply community participatory-based principles. These principles include building on the strengths and resources of the community; mobilizing collaborative, equitable partnerships; and fostering colearning and capacity building among all partners. These underlying principles are necessary across all phases of PSE interventions to create and foster long-term multisector partnerships that promote and sustain activities (11). In Hidalgo County, residents now have access to additional opportunities for active living, and coalition members continue to collaborate and secure resources to expand physical activity infrastructure. Moreover, coalition members have expanded activities outside the original 4 intervention communities.

Communities with similar challenges related to physical activity infrastructure gaps in the built environment should consider PSE-focused interventions that involve community members at the outset. Meaningful community engagement can help public health practitioners better understand community challenges and formulate solutions that effectively address systemic barriers. This engagement is especially important in advancing policy efforts that require substantial support from the community and political will from elected officials.

Although PSE-focused interventions can enhance and expand the reach of public health initiatives, sustaining such efforts requires significant resources related to community involvement, technical expertise from subject matter experts, and organizational backbone support from public health partners. In Hidalgo County, Texas A & M University staff members continue to engage with coalition members, albeit in a much more limited capacity. Further research is needed to explore how PSE strategies can be broadly incorporated into traditional community health programming to maximize impact, reach, and sustainability of public health interventions.

**Box. Summary of actions and time to increase access and physical activity in Hidalgo County, Texas**

2015 — Working on Wellness (WoW) coalitions were established to increase access to physical activity and healthy food in Hidalgo County, Texas. Coalition members and partners identified bicycle infrastructure as key to promoting and increasing physical activity access. Collaboration began with community members and governmental partners to address gaps in bicycle infrastructure.

2016 — Five miles of bicycle lanes were installed in Weslaco.

2017 — The Weslaco Master Trails Plan was adopted. Coalition members partnered with the Hidalgo County Metropolitan Planning Organization to implement the Bicycle Friendly Business program.

2018 — Weslaco installed an additional one-half mile of bicycle lanes. WoW staff and coalition partners collaborated with the Hidalgo County Metropolitan Planning Organization to implement the Hidalgo County Walkability Team Action Plan.
