# Erratum, Vol. 16, September 12 Release

**DOI:** 10.5888/pcd16.190125e

**Published:** 2019-10-17

Figure 2 of the Research Brief “Expanding Bicycle Infrastructure to Promote Physical Activity in Hidalgo County, Texas” was incorrectly captioned. The original caption indicated that the photograph was taken in Edinburg, Texas, by Robert Deleon. The photograph was actually taken in McAllen, Texas, by Robert DeLeon. The correction was made on our website on October 3, 2019, and appears online at https://www.cdc.gov/pcd/issues/2019/19_0125.htm. We regret this error and any confusion it might have caused.

